# Patterns of Occurrence of Sharks in Sydney Harbour, a Large Urbanised Estuary

**DOI:** 10.1371/journal.pone.0146911

**Published:** 2016-01-29

**Authors:** Amy F. Smoothey, Charles A. Gray, Steve J. Kennelly, Oliver J. Masens, Victor M. Peddemors, Wayne A. Robinson

**Affiliations:** 1 NSW Department of Primary Industries, Sydney Institute of Marine Science, Mosman, NSW, Australia; 2 WildFish Research, Grays Point, NSW, Australia; 3 IC Independent Consulting, Cronulla, NSW, Australia; 4 School of Environmental Sciences, Charles Sturt University, Thurgoona, NSW, Australia; University of Sydney, AUSTRALIA

## Abstract

Information about spatial and temporal variability in the distribution and abundance of shark-populations are required for their conservation, management and to update measures designed to mitigate human-shark interactions. However, because some species of sharks are mobile, migratory and occur in relatively small numbers, estimating their patterns of distribution and abundance can be very difficult. In this study, we used a hierarchical sampling design to examine differences in the composition of species, size- and sex-structures of sharks sampled with bottom-set longlines in three different areas with increasing distance from the entrance of Sydney Harbour, a large urbanised estuary. During two years of sampling, we obtained data for four species of sharks (Port Jackson, *Heterodontus portusjacksoni*; wobbegong, *Orectolobus maculatus*; dusky whaler, *Carcharhinus obscurus* and bull shark, *Carcharhinus leucas*). Only a few *O*. *maculatus* and *C*. *obscurus* were caught, all in the area closest to the entrance of the Harbour. *O*. *maculatus* were caught in all seasons, except summer, while *C*. *obscurus* was only caught in summer. *Heterodontus portusjacksoni* were the most abundant species, caught in the entrance location mostly between July to November, when water temperature was below 21.5°C. This pattern was consistent across both years. *C*. *leucas*, the second most abundant species, were captured in all areas of Sydney Harbour but only in summer and autumn when water temperatures were above 23°C. This study quantified, for this first time, how different species utilise different areas of Sydney Harbour, at different times of the year. This information has implications for the management of human-shark interactions, by enabling creation of education programs to modify human behaviour in times of increased risk of potentially dangerous sharks.

## Introduction

Knowledge of how organisms are spatially and temporally distributed is fundamental to understanding their ecology and population dynamics [1]. Many studies, across a range of environments, have shown that most organisms have highly variable and interactive patterns of abundance through space and time [2–4]. Increasing attention has been given to the importance of examining variation at a hierarchy of different spatial and temporal scales when measuring abundance of organisms [5, 6]. However, estimating patterns of distribution and abundance of sharks across a variety of spatial and temporal scales can be difficult due to species-specific patterns of movements, migrations [7] and some species exhibiting relatively solitary behaviour making their occurrences, in any place and time, sporadic and rare. Nevertheless, prior to commencing large-scale, long-term studies monitoring populations of sharks in this region, it is necessary to identify relevant scales of variation and how common any patterns of variation are across different spatial scales.

Many species of sharks are considered apex predators with the potential to alter community-structure either directly or indirectly [8–10]. Many populations of sharks have, however, declined throughout the world due to the alteration and degradation of coastal habitats and increased fishing [11–13]. Knowledge of characteristics of populations, such as size structures, segregation by sex and maturity, where and what habitats sharks occupy and over what time scales, together with estimates of abundances are particular areas of interest and are vital pre-requisites to the management of shark populations and consequent conservation. Similarly, such understanding will also assist with reducing risk of negative human-shark interactions through enabling creation of education programs to modify human behaviour in high risk zones [14] and/or be more target-specific in the implementation of any shark mitigation strategies.

Estuaries are diverse and productive ecosystems providing highly dynamic environments where species distributions and abundances are often a result of a complex interplay of physical (e.g. temperature, salinity, turbidity, tidal movements) and biological processes (e.g. reproduction, mating and feeding). Studies around the world have highlighted the importance of estuaries [15–20] in supporting assemblages of sharks, particularly juveniles, yet there is a paucity of such information from south-eastern Australia.

Sydney Harbour is one of the largest and most biological diverse estuaries in the world. Coupled with this, it is Australia’s busiest, most industrialised and urbanised estuary playing a significant economic, social and environmental role for the city of Sydney, housing 4.8 million people [21, 22]. Consequently, this system is subjected to a range of anthropogenic impacts (see review by [23]). Although Sydney Harbour has a diverse assemblage of fishes [21, 24, 25], the majority of studies have largely been done on intertidal and/or subtidal invertebrate assemblages (see review by [22]). At present, species-composition of sharks and their patterns of distribution and abundance across spatial and temporal scales are largely unknown. Nevertheless, catch and effort data from the New South Wales shark meshing program reported seasonal trends in catch of sharks [26]. Catches of hammerhead sharks (*Sphyrna* spp.), whaler sharks (*Carcharhinus* spp.) and grey nurse sharks (*Carcharhinus taurus)* peaked in Austral summer months. Tiger sharks (*Galeocerdo cuvier*) had reduced catches during the cooler months (September-December), in contrast, white sharks (*Carcharodon carcharias*) and Port Jackson sharks (*H*. *portusjacksoni*) were predominately caught in spring [26]. Further, the abundance of *O*. *maculatus* was found to be greater in spring and summer than autumn and winter in areas around Sydney [27, 28].

Shark bites in Australia have garnered substantial political interest and led to active shark bite mitigation strategies being implemented in Queensland, New South Wales and West Australia [29–31]. Most shark interactions have occurred in ocean waters, with a distinct seasonal peak in shark interactions with 71% of bites occurring between November and April [32]. Considering the heavy recreational use of urbanised estuaries within NSW, there have been relatively few serious shark bites in these waterways. However, following a particularly severe interaction in Sydney Harbour during the summer of 2008/9, the NSW government requested more information on shark abundance and distribution in this iconic waterway. This study was, therefore, established to examine differences in the composition of species, size- and sex-structures of sharks over a two year period in Sydney Harbour. We used a hierarchical sampling program involving bottom-set longlines with the aim to test whether: (i) there would be temporal variation at the largest scale of three months (seasons), but not at smaller temporal scales (i.e. weeks and months), (ii) differences in abundance of shark species among areas in Sydney Harbour would be species dependent, (iii) patterns would be consistent among years and (iv) significant differences in abundances of sharks across seasons and/or areas in the estuary, if present, could be explained by a relationship with water temperature. Low abundances of sharks in this study restricted the number of testable parameters precluding testing of hypotheses (i) to (iii). Alternatively, we used an information-theoretic approach, fitting statistical models under hypothesis (iv), considering whether the area in Sydney Harbour and/or water temperature (as a surrogate for seasonal effects) could be used to model the probability of shark-capture.

## Methods

### Ethics statement

Sampling was done under NSW Agriculture Animal Care and Ethics approval (Permit 07/08) and in accordance with New South Wales Department of Primary Industries Research Permit Section 37 (PO1/0059A-2.0).

### Study location

**Fig 1:**
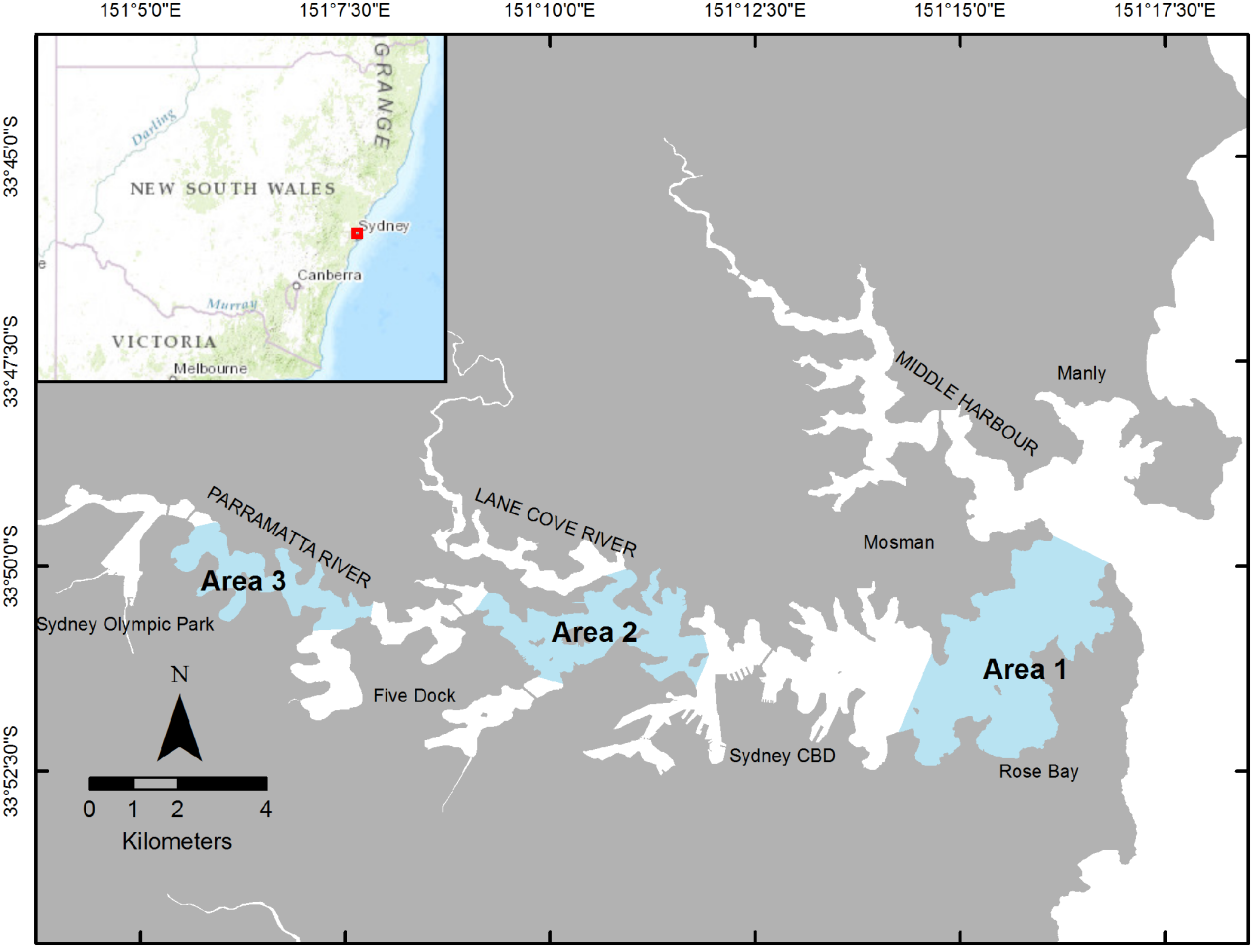
Map of sampling areas in Sydney Harbour, Australia. Area 1 represents the entrance area, area 2 represents the central location and area 3 represents the upper location.

Sydney Harbour (~ 33°51’S, 151°14’E, Fig 1) is a large, deep, drowned river valley approximately 30 km long, 3 km at its widest point and covers an area of 55 km^2^ with numerous tributaries and waterways [33]. The morphology of the seabed is complex and irregular with a series of deep holes up to 47 m deep, however, most embayments are relatively shallow (< 15 m). The estuary is fully tidal and has a relatively small freshwater flow from two rivers, the Parramatta and Lane Cove Rivers [33]. Salinity reflects marine conditions (~ 35 ppt) but declines after heavy rainfall when there is often a surface layer of fresh water that can extend up to half the length of the Harbour.

### Sampling design and methods

A hierarchical sampling design was used to examine differences in the diversity and abundance of sharks among areas and times of sampling within Sydney Harbour over two years (2010–2012). Three areas with increasing distance from the harbour entrance (entrance, central and upper) were separated by several kilometres from east (the entrance) to west (the upper reaches) in Sydney Harbour (Fig 1). Each area was sampled at night, over two consecutive weeks, in two consecutive months across all four seasons each year. Therefore, six randomly selected nights were sampled over a two-week period in a month. On each night of sampling, four replicate set-lines were interspersed haphazardly across one of the areas (ranging in depth from 1.5 to 29 m) with greater than 200 m spacing between each set-line. To investigate the consistency in patterns between years, sampling was done over two consecutive years. We recorded various water properties (temperature, salinity, dissolved oxygen, pH and turbidity) just below the surface and just above the substratum at each of the four replicate set-lines, at each time of sampling, as possible co-variables for analyses. However, preliminary analyses showed that surface water temperature was a better co-variate than bottom water temperature, or the other recorded variables. Here, we only presented results for surface water temperature for brevity and given, we predicted that temperature would explain the observed patterns of distribution and abundance of sharks.

Bottom-set longlines were chosen above other fishing-methods (e.g. drum-lines) because they: (1) are known to be successful in catching various species of large sharks targeted commercially in NSW [34], (2) provided a large amount of sampling-effort, and (3) were least hazardous to other vessels within the heavily congested Harbour. Set-lines consisted of a 200 m long, bottom-set mainline consisting of 7 mm braided lead-core rope anchored at each end. There were 15 snoods per set-line spaced 13 m apart. Snoods were 3 m long and made of 3 mm plastic coated stainless steel wire trace; breaking strain of 400 lb. Each snood was connected to the mainline via a shark clip and had a 16/0 tuna circle hook baited with half a frozen sea mullet (*Mugil cephalus*). A burley canister was attached to both of the surface floats, at each end of the set-line, with a predefined and consistent frozen mixture made from 500 g of minced Australian pilchard (*Sardinops sagax*), 500 g of chicken layer pellets and 250 ml of tuna oil. The set-lines were bottom-set two hours before dusk, soaked for two hours and retrieved. Captured sharks < 1.4m total length were brought aboard the research vessel, identified, measured (precaudal, fork and total), sexed, dart-tagged, and released. Larger sharks (> 1.4 m total length) were brought alongside the research vessel, where they were identified, tail-roped and inverted to induce tonic immobility. The sex and lengths (precaudal, fork and total) of each shark were recorded. All released sharks were dart-tagged to enable rapid identification of recaptures. All bull sharks (*C*. *leucas)* were acoustically tagged with a 16 mm Vemco acoustic transmitter during this procedure for use in a separate experiment with the entire handling process taking less than 15 minutes. The hook was removed prior to release.

### Statistical analysis

The anticipated statistical measure of abundance used was catch per unit effort (CPUE), defined as the number of sharks caught per 60 hooks per night (2 hours of sampling) in the randomly selected area. There was a large number of zeroes in the data-set. Thus, to moderate the statistical limitations resulting from small abundances, we aggregated the catch data to higher spatial and temporal scales prior to analysis. This limited our ability to test hypotheses (i) to (iii). Further, the data were analysed only for the two most dominant species. *C*. *leucas* consisted of 24 records, one replicate per area, per season, per year, and *H*. *portusjacksoni* had 8 replicates from the entrance, in both years. Captures of *O*. *maculatus* and *C*. *obscurus* were low and were excluded from statistical analysis. To ensure independence of the data, recaptured animals were excluded from data analysis. We investigated temporal variability in the relative abundances of sharks by using the average surface water temperature on each sampling occasion in each season, as a covariate. This technique used fewer parameters in the analysis, helping to moderate statistical limitations of the data-set.

We used an information theoretic approach to consider several candidate generalised linear models to explain variation in captures of *C*. *leucas* between sampling areas and the relationship with water temperature. We considered this approach appropriate because of the sparse nature of our data and because water temperature is an observed rather than controlled co-variate; with different ranges in the different areas. We considered five models to explain patterns of captures of *C*. *leucas* including; area and water temperature, fitted in separate or combined Poisson regression models, and; zero—inflated Poisson regression models with temperature modelled as a possible zero inflation factor, with and without area. In the latter two models, the zero inflation factor was fitted as a logit-link binomial variable predicting presence or absence of the species. Goodness of fit for all models was assessed using the residual deviance/residual df where values > 1 indicated over-dispersion and values < 1 under-dispersion of the data [35]. The Poisson regression models assessed whether the abundance of *C*. *leucas* could be predicted by knowing: i) the area of the harbour where sampling occurred or, ii) the temperature of the water during sampling or, iii) both the area and temperature during sampling. The zero inflation models assessed whether the occurrence of *C*. *leucas*, could be predicted by water temperature during sampling, iv) with and, v) without area (to predict abundance) included in the models. This approach was used because we predicted that *C*. *leucas* may have different abundances across the different areas of the harbour and that water temperature may be a useful predictor of the time of year that sharks occur in Sydney Harbour. The corrected Akaikes Information Criteria (AICc) values [36] as recommended by [37] when sample sizes are small, was used to compare all models. All models that had lower AICc values than the unconditional model (no factors) were used to predict captures of *C*. *leucas* in Sydney Harbour using AICc as weights. When models included area as an effect we used Wald 95% confidence intervals and Wald Chi square test of the parameter estimates to assess whether there were differences in captures of *C*. *leucas* among areas.

*Heterodontus portusjacksoni* never occurred in the central or upper areas within the Harbour and thus only the eight replicates from the entrance were analysed. Models were evaluated to assess whether temperature was able to predict seasonal captures of *H*. *portusjacksoni* using the Poisson model, with and without the zero inflation effect. All analyses were carried out using the Genmod procedure in [38].

## Results

During the two years of sampling, we fished 85 nights, deployed 340 set-lines with 5100 hooks and caught a total of 45 sharks comprising of four species (Table 1). Captures occurred across all areas within Sydney Harbour, but most sharks were captured closest to the mouth of the Harbour, with some sharks caught in each of the central and upper areas only in the periods of January to March (Table 1).

**Table 1:** Total number of sharks caught in each season across the three areas of sampling in Sydney Harbour between 2010–2012.

	Autumn	Winter	Spring	Summer
*(i) Carcharhinus leucas*				
Entrance	0	0	0	4
Central	2	0	0	4
Upper	0	0	0	2
*(ii) Heterodontus portusjacksoni*				
Entrance	0	18	8	1
Central	0	0	0	0
Upper	0	0	0	0
(iii) *Orectolobus maculatus*				
Entrance	3	1	1	0
Central	0	0	0	0
Upper	0	0	0	0
(iv) *Carcharhinus obscurus*				
Entrance	0	0	0	1
Central	0	0	0	0
Upper	0	0	0	0

During the first year of sampling, four male and one female *C*. *leucas* were caught, ranging in size from 2.15 to 3.12 m (TL). In the second year of sampling, six males and one female were caught (2.34–3.02 m TL). *C*. *leucas* were caught in all three areas and captured in the period January to April (Table 1). Three models were useful for predicting patterns of abundance of *C*. *leucas* in Sydney Harbour and all included surface water temperatures (Table 2). The model that included only area was not a good fit (over-dispersed and increased AICc, Table 2), however, when included with temperature in a Poisson regression, area was a useful predictor (Table 2). The model using just temperature as a zero inflation Poisson predictor was the best individual model and contributed 60% of the weight in the overall prediction model. When the Poisson regression model including temperature + area was applied, capture of *C*. *leucas* were significantly greater in the entrance (χ^2^ = 5.11, df = 1, p < 0.05) and central (χ^2^ = 7.46, df = 1, p < 0.01) locations than the upper location (Table 2).

**Table 2:** Summaries of models for captures of (i) *Carcharhinus leucas* in three areas of sampling and (ii) *Heterodontus portusjacksoni* in the entrance of Sydney Harbour and their relationships with water temperature.

Model	AICc	AICc weight	No. Parameters	Residual df	Residual deviance ÷ df	Poisson Regression parameters				ZiP regression Parameters	
						*b*_*0*_	*b*_*T*_	*b*_*E*_	*b*_*C*_	*b*_*0Z*_	*b*_*TZ*_
(i) *Carcharhinus leucas*											
1 Captures = Temperature (ZiP)	37.3	0.60	2	21	1.4	0.72				20.58	-0.89
2 Captures = Area (Poi) + Temp (Poi)	38.8	0.28	3	20	0.8	-23.57	0.93	2.11*	2.48*		
3 Captures = Temperature (Poi)	40.6	0.12	1	22	1.0	-12.16	0.51				
Unconditional Model	55.5		0	23	1.7						
Captures = Area (Poi)	58.5	0.00	2	21	1.8						
Captures = Area (Poi) + Temp (ZiP)		0.00	5	19	Poor fit						
(ii) *Heterodontus portusjacksoni*											
1 Captures = Temperature (Poi)	26.8	1.00	1	6	0.7	10.61	-0.52				
2 Captures = Temperature (ZiP)	47.2	0.00	2	5	1.4	1.69				-16.88	0.78
Unconditional Model	56.9		0	7	5.4						

(Poi) indicates the predictor variable is fitted using a Poisson regression and (ZiP) using a zero-inflated Poisson regression. *b*_*0*_ = constant, *b*_*T*_ = temperature, *b*_*E*_ = Harbour entrance, *b*_*C*_ = Harbour central, *b*_*0Z*_ = Constant ZiP Model, *b*_*TZ*_ = temperature ZiP model and *p < 0.05.

**Fig 2:**
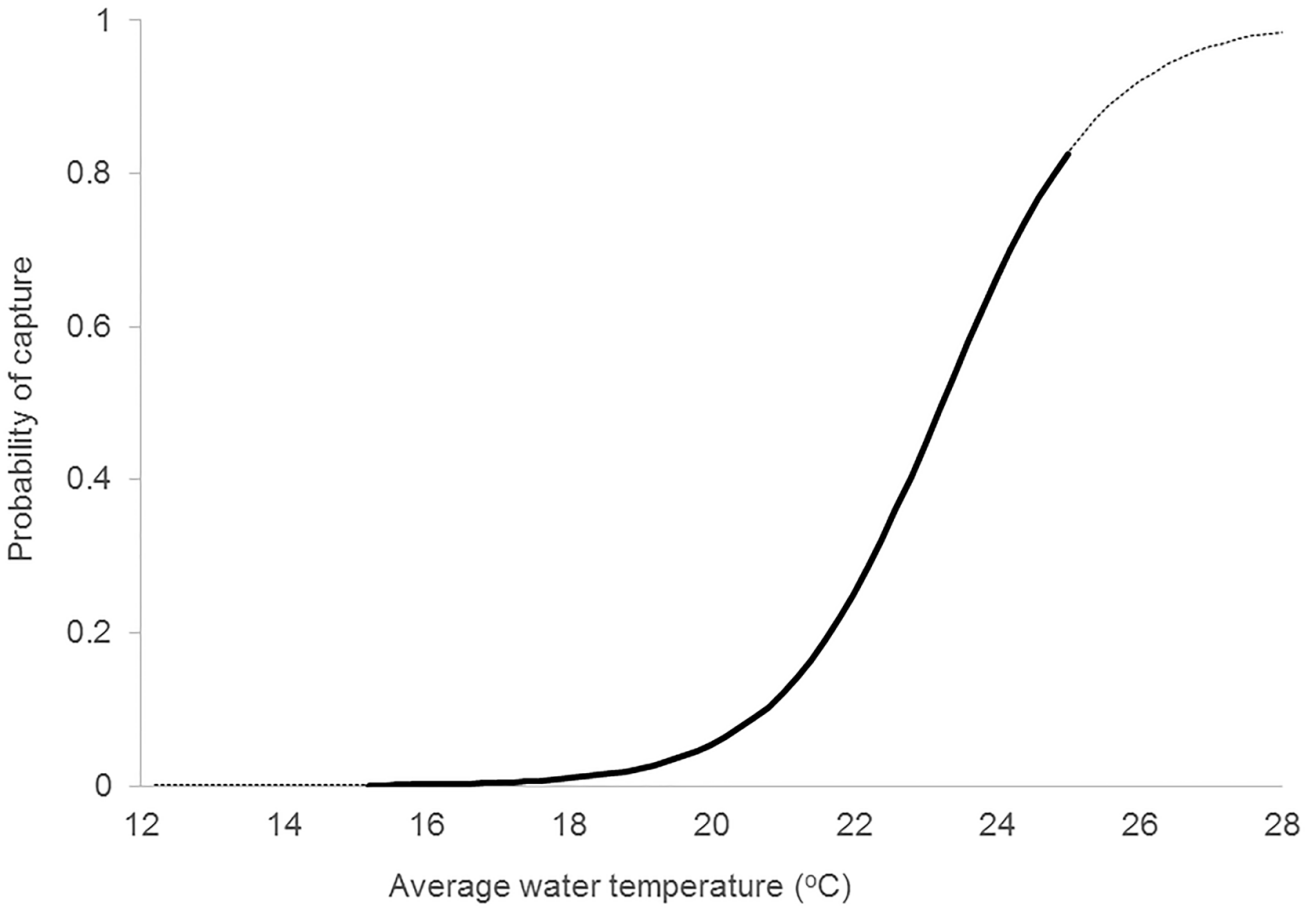
Modelled probability of capture of *Carcharhinus leucas* in Sydney Harbour in relation to monthly average surface water temperature (°C). Solid line is the range of data collected, dotted line is extrapolation.

**Fig 3:**
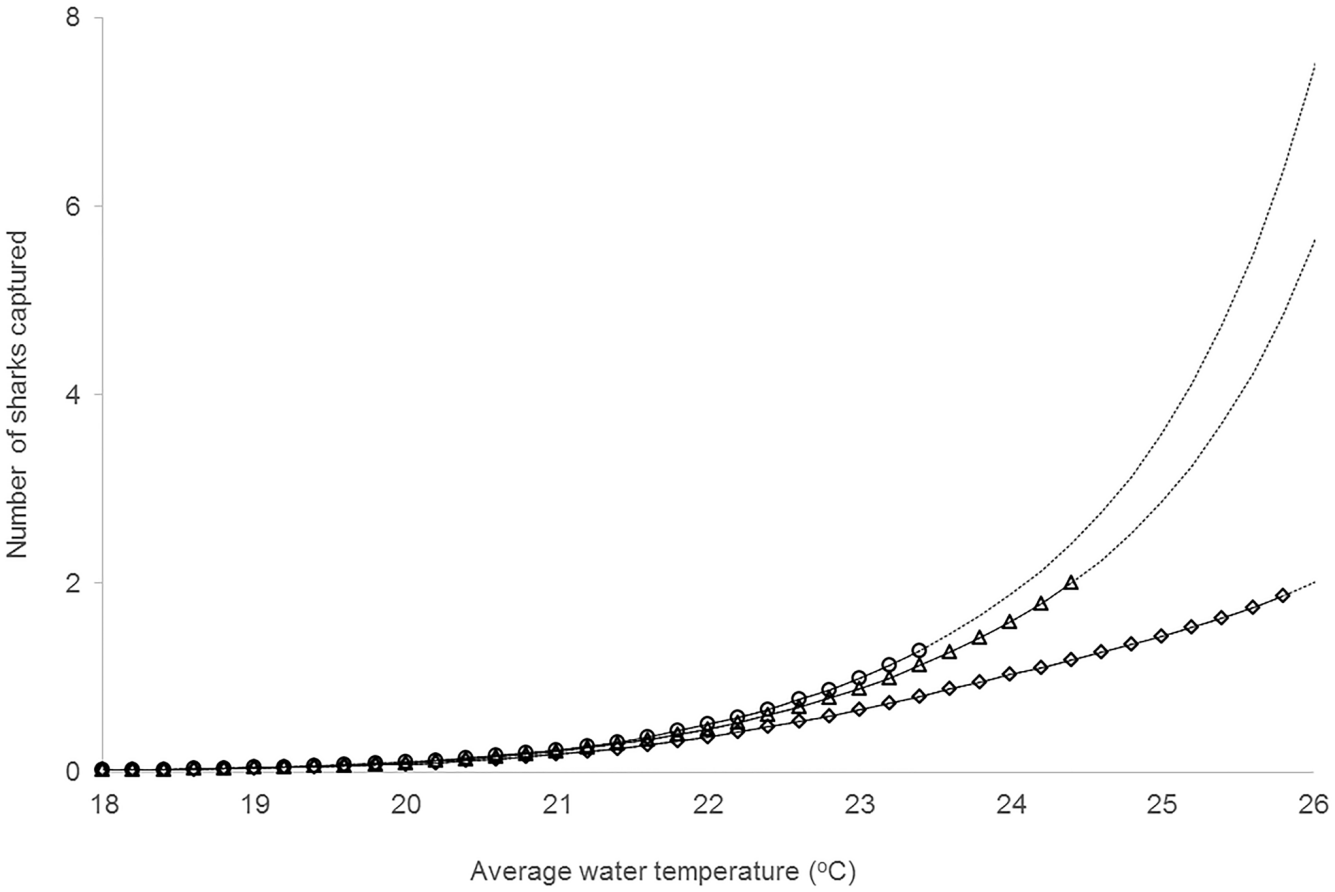
Model averaged predicted relationship between average surface water temperature (°C) and captures of *Carcharhinus leucas* in three areas in Sydney Harbour (○ = entrance, Δ = central, ◊ = upper). Solid lines indicate the range of data collected, dotted lines are extrapolation.

The model including only temperature as a zero-inflation Poisson predictor showed some over-dispersion (Deviance/df = 1.4, Table 2). Nevertheless, the AICc comparisons, showed this model had good utility for prediction, and by extracting the model’s zero inflation component we predict that in Sydney harbour, *C*. *leucas* were very unlikely to be caught (P|capture| < 0.02) when water temperature is below 19°C; being more likely to be caught than not P|capture| > 0.50) at about 23.2°C (Figs 2 and 3). The over-dispersion was not evident in the other two useful models (Table 2).

**Fig 4:**
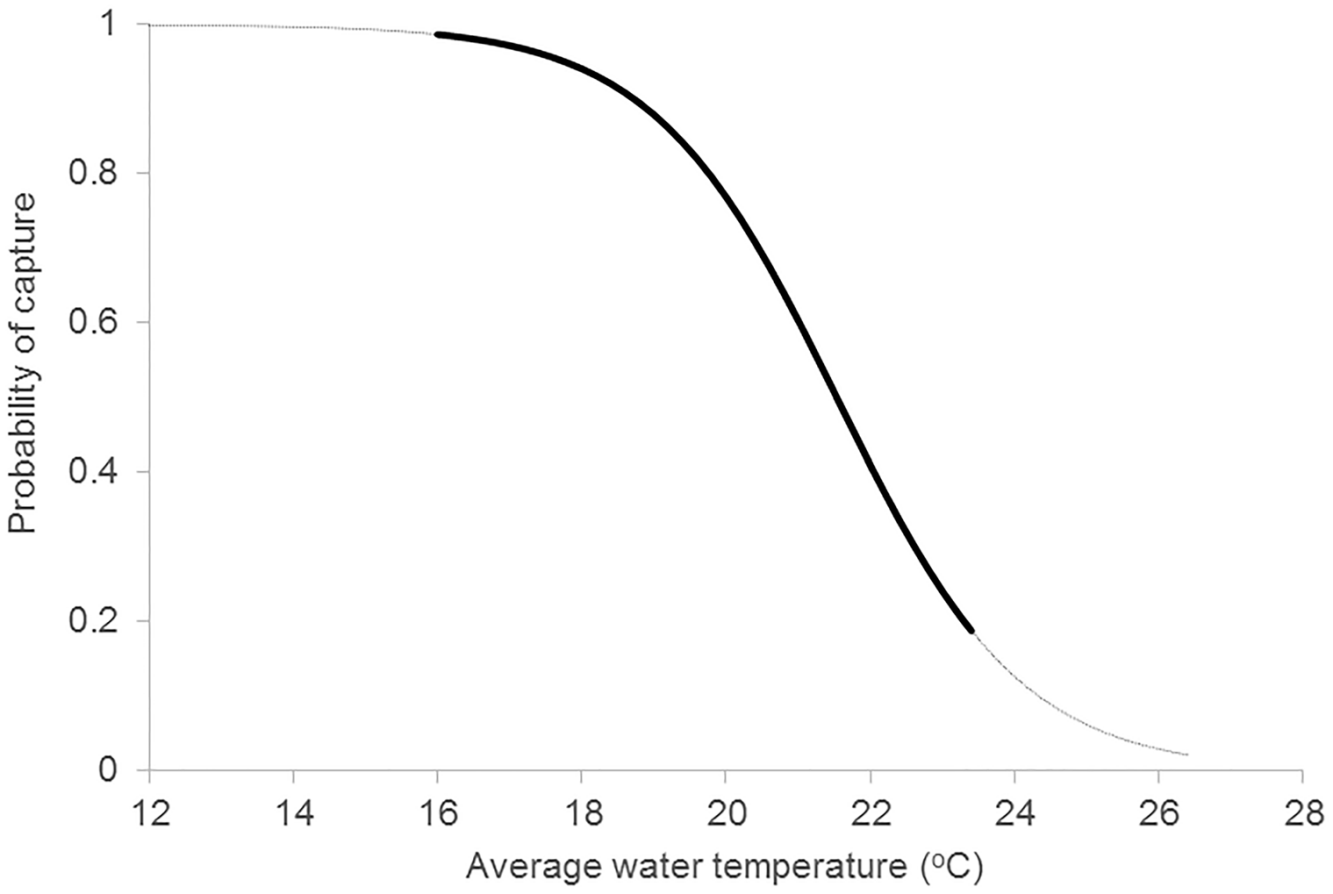
Modelled probability of capture of *Heterodontus portusjacksoni* in the entrance area of Sydney Harbour in relation to monthly average surface water temperature (°C). Solid line is the range of data collected, dotted line is extrapolation.

**Fig 5:**
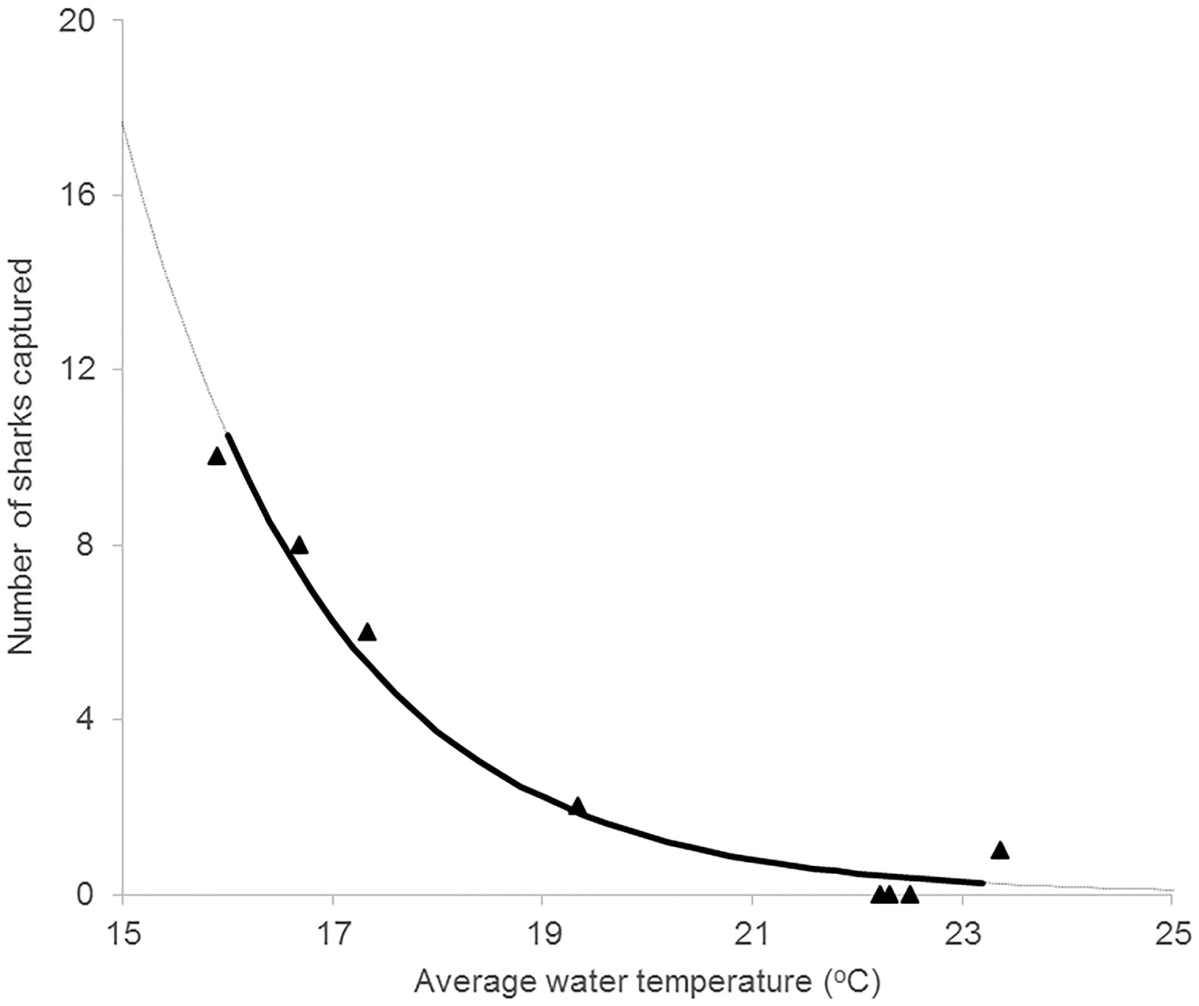
Model predicted relationship between average surface water temperature (°C) and captures of *Heterodontus portusjacksoni* in the entrance area of Sydney Harbour. Solid line is the range of data collected, dotted line is extrapolation. Solid symbols are actual data.

*Heterodontus portusjacksoni* were the most numerous species, yet they were only recorded in the entrance area and mostly between July to November each year (Table 1). During the first year of sampling, we caught 12 females and one male (0.95 m TL). These individuals ranged in size from 0.95 to 1.20 m (TL). Similarly, in 2011–2012, we caught 13 females and one male (1.04 m TL), ranging in size from 1.02 to 1.27 m. The zero—inflated Poisson model was a useful model to predict the relationship between *H*. *portusjacksoni* presence and water temperature (Table 2). It showed that in the entrance area, *H*. *portusjacksoni* were unlikely to be caught (P|capture| < 0.02) when water temperature was above 26.5°C and more likely to be caught than not (P|capture| > 0.50) when the water temperature was below 21.5°C (Figs 4 and 5). The regular Poisson regression model was deemed more valuable than the Zero-inflated model when predicting capture of *H*. *portusjacksoni* and retained all the weight in the prediction (Table 2).

Only one *O*. *maculatus*; male 1.49 m total length, was caught during the first year of sampling, whereas four *O*. *maculatus*; three females and one male (1.01–1.37 m TL), were caught during the second year of sampling. All *O*. *maculatus* were caught in the area closest to the Harbour entrance (Table 1). Only one juvenile (1.02 m TL) female, *C*. *obscurus* was recorded over the entire two year sampling regime, and that was in entrance area during summer (Table 1).

## Discussion

This study is the first to quantify the species composition of sharks in Sydney Harbour and to examine their spatial and temporal variability using a rigorous, hierarchical sampling design. Despite the relatively low catch-rates, our data indicated species-specific patterns in abundance that were consistent between years. *H*. *portusjacksoni* was the dominant species caught in Sydney Harbour being consistently more abundant in the entrance area and caught only during Austral winter and spring, when water temperature was below 21.5°C. The likely explanation for this pattern is associated with mating. Studies have shown that adult *H*. *portusjacksoni* aggregate on shallow coastal rocky reefs in New South Wales between July and November to mate [39, 40] and females deposit their egg capsules within rocky crevices between August and October [39]. For this reason, one would predict that female *H*. *portusjacksoni* would only be found in areas of rocky reef. Although this study did not quantify the types and relative abundance of different habitat-types in each of the three areas, observations indicate that there was a greater proportion of rocky reef in the entrance area than the other areas in Sydney Harbour, providing evidence to support the observed pattern of *H*. *portusjacksoni* distribution. The apparent dispersal of *H*. *portusjacksoni* from Sydney Harbour during the warmer months of November to June should be further investigated, possibly via acoustic telemetry studies.

*Carcharhinus leucas* showed large variability in catches, but there was an overall trend through time indicating they were more numerous in summer and autumn than winter and spring. Similar to the patterns observed here, Cliff and Dudley [41] reported a peak in catch of *C*. *leucas* in bather-protection nets off the coast of South Africa during December, with a decline during winter and spring. Increased catches of tropical and subtropical species of sharks in summer and decreased catch-rates in winter have been related to changes in water temperature. Heithaus [42] and Wirsing et al. [43] found that *G*. *cuvier* catch-rates in the Eastern Gulf of Shark Bay, Western Australia were consistently greater during the warm season (September-May), when surface water temperature was greater than 20°C, than during the colder months (June-August). In Sydney Harbour, water temperature also appeared to influence the rate of capture of *C*. *leucas* when surface water temperature was greater than 23.2°C. Temperature may therefore directly or indirectly influence the occurrence of *C*. *leucas*.

These temporal patterns may be linked to movements of the warm waters of the East Australian Current (EAC) [44]. In order to minimise energetic demands of key metabolic and physiological processes [45], *C*. *leucas* may use the strong EAC to migrate southwards during summer and return northward during winter, when the current is weakest and inshore waters are cooler. Large-scale migrations of *C*. *leucas* on the east coast of Australia were examined by Heupel et al. [7], yet the drivers for these movements to and from coral reef regions still remain unknown. Similar seasonal patterns of distribution and movement for *C*. *leucas* have been reported along the east coast of South Africa [46], where *C*. *leucas* migrated north to warmer latitudes during austral winter and spring and southward movement into more temperate latitudes during summer. Similar patterns of temperature mediated movement have been found for other elasmobranchs. Couturier et al. [47] reported seasonal patterns of distribution and movement for the manta ray, *Manta alfredi* along the east coast of Australia and found that same-site visitation was most likely related to changes in sea water temperature, current flow and food abundance. Movements of juvenile sharks have also been reported to be regulated by temperature, with juvenile blacktip (*C*. *limbatus*) and sandbar sharks (*C*. *plumbeus*) in south-east USA migrating south to warmer waters during colder months of the year [48, 49].

As sharks are poikilothermic, it is unlikely that water temperature per sê regulates their movements. Movement in apex predators is generally considered to be driven by food and/or reproductive requirements. In the case of movements of *C*. *leucas* into Sydney Harbour, it is likely that water temperature may be indirectly affecting the occurrence of prey species in this waterway, such that *C*. *leucas* could be using Sydney Harbour as a feeding ground. During summer and autumn, yellowtail kingfish (*Seriola lalandi*), Australian bonito (*Sarda australis*), frigate mackel (*Auxis thazard*) and mackerel tuna (*Euthynnus affinis*), among others, were observed schooling within Sydney Harbour (pers. obs.), all of which may be potential prey species of *C*. *leucas*. Prey abundance and patterns of movement are believed to be one of the main factors determining the distribution of a number of shark species [50–53]. An overlap in abundance and distribution of seven shark and 45 prey species has been reported in Florida Bay [54]. However, they found that catch-rates of shark were not directly related to catch-rates of teleosts at small spatial scales (i.e. individual sampling sites) [54]. That said, Hammerschlag et al. [55] found very little overlap in the habitat-use of *C*. *leucas* and a potential prey species, Altantic tarpon (*Megalops atlanticus*) in southern Florida. However, by simultaneously tracking the movements of a predator (broadnose sevengill shark, *Notorynchus cepedianus*) and five of its known prey in Norfolk Bay, Tasmania, Barnett and Semmens [56] found that the predator and its prey showed high spatial overlap and similar patterns of habitat-use, suggesting that *N*. *cepedianus’* seasonal migrations are associated with exploiting the seasonal abundance of its prey. Further fine-scale research is required in Sydney Harbour to elucidate the role of prey in determining shark abundance and distribution.

Another possible explanation for the observed pattern of catch is that *C*. *leucas* are more numerous in warmer months in Sydney Harbour as this estuary could possibly be a site for mating. Although rates of catch were low, our data indicated a population consisting mostly of adult *C*. *leucas*. Mating behaviour in elasmobranchs is poorly documented with most observations coming from captive animals [57]. Pratt and Carrier [58] documented adult nurse sharks (*Ginglymostoma cirratum*) displaying site fidelity for mating purposes in Florida by returning to the same site annually for males and every second year for females. Limited information is available about the reproductive biology of *C*. *leucas*. It is suggested that gestation is 10–11 months, yet the frequency of its reproductive cycle is still unknown [59]. Observations made of the catch of commercial shark fishers in the northern rivers of New South Wales, suggest that neonate *C*. *leucas* have the presence of an umbilical cord, or umbilical slit, in November. Thus, if gestation is 10–11 months, this would imply that mating occurs in January, coinciding with the peak period of abundance of sexually mature *C*. *leucas* in Sydney Harbour detected in this study. Although one shark was captured with apparently fresh mating scars, further research, with a greater sample size is required to determine whether Sydney Harbour is used for mating by *C*. *leucas*.

Interestingly, the absence of neonates in our data suggests that Sydney Harbour is not a pupping ground. *C*. *leucas* has large litter sizes, with females giving birth to 1–13 pups [59]. If Sydney Harbour was functioning as an area for pupping, then one would predict that pups should have been caught in relatively large numbers. Similarly, the lack of juveniles in the catch implies that Sydney Harbour does not constitute a nursery ground. Small *O*. *maculatus*, *H*. *portusjacksoni* and a juvenile *C*. *obscurus* were caught, implying that the gear-type is not responsible for the absence of neonates. Further, we consistently caught neonates and young-of-the-year *C*. *leucas* using this method in the northern rivers of NSW, as do commercial fishers (Smoothey, unpublished data), implying that the gear-type is not responsible for the absence of neonates in the catches in Sydney Harbour. In other parts of the world, where studies have identified pupping areas for *C*. *leucas*, substantial numbers of neonates and young of the year *C*. *leucas* are frequently caught using bottom-set longlines with hook-size similar to that used in this study [15]. We believe, therefore, it unlikely that our fishing method led to bias in the data, but that these data provide evidence for size-segregation occurring in this population. Size-segregation in habitat-use is commonly found in *C*. *leucas* [20, 60–62] and other chondrichthyans [63, 64], with neonates living in nursery areas for weeks, months or years [65].

Overall relative abundances of *C*. *leucas* in this study (as estimated using catch-rates from standardized fishing) were low, as one may expect from a relatively rare, mobile species at the southern extent of its distribution [7, 59]. Nevertheless, because of the levels of replication and hierarchical design used, we believe these were adequate to distinguish between sampling zeros, not finding a species and structural zeros, a true measure of absence [66]. Further, rates of catch are reflective of abundance and not expected to be a limitation of sampling gear with many studies previously using this method of fishing to target various species of sharks in Australia [34, 67–71] and elsewhere [15, 19, 72–74]. Although bottom-set longlines may tend to catch more demersal species of sharks and hence could be considered to represent a bias to reduced capture of pelagic species, like the great white (*C*. *carcharias*) and tiger sharks (*G*. *cuvier*), several studies elsewhere have successfully captured these two species using bottom-set longlines [34, 75, 76]. Moreover, although Heithaus et al. [72] indicated there may be species-specific effects of bait-type on shark catch-rates, our study used *M*. *cephalus*, a common prey species for sharks off eastern Australia. *M*. *cephalus* has been successfully used in local commercial shark fisheries catching numerous species of different families [34, 77]. The lack of capture of *C*. *carcharias* and *G*. *cuvier* therefore most likely reflects the rare occurrence of these species in Sydney Harbour, rather than a sampling bias.

Another assumption of this study is that rates of catch effectively measure the abundance of sharks in Sydney Harbour and that lower catch-rates indicate movements of sharks out of the study area. It is worth noting that low rates of catch may reflect lower feeding rates rather than actual changes in abundance. Free-swimming *C*. *leucas* (tagged and untagged) were only sighted in Sydney Harbour during warm months and no detections were made of acoustically tagged sharks during cold months [Smoothey, unpublished data]. Based on this line of evidence, catch-rates in Sydney Harbour are believed to be a true reflection of shark abundance.

Given the important role played by sharks in marine ecosystems, their world-wide over-exploitation and the general increase of human-shark interactions [14, 32], there is much interest in their conservation and management. Nevertheless, reliable empirical knowledge about their patterns of distribution and abundance upon which effective management decision can be based are generally lacking. This is particularly important for understanding and potentially managing shark-human interactions, especially in highly populated estuaries like Sydney Harbour. This study has shown, through the use of a hierarchically-designed and well-replicated survey, that one can quantify the abundances of these mobile, solitary organisms in such estuaries and identify the possible influence that co-variables such as seasonal water temperature can have on their presence. Future work focused on better understanding the movements of sharks principally responsible for unprovoked bites, for example *C*. *leucas* in this system, will hopefully increase our understanding of shark biology and contribute to objective assessment of the risk of shark bites.
